# Driving Environment Perception Based on the Fusion of Vehicular Wireless Communications and Automotive Remote Sensors

**DOI:** 10.3390/s21051860

**Published:** 2021-03-07

**Authors:** Minjin Baek, Jungwi Mun, Woojoong Kim, Dongho Choi, Janghyuk Yim, Sangsun Lee

**Affiliations:** 1 Department of Electronics and Computer Engineering, Hanyang University, Seoul 04763, Korea; mjbaek@hanyang.ac.kr (M.B.); moonjw91@hanyang.ac.kr (J.M.); cdh5375@hanyang.ac.kr (D.C.); jhyim@hanyang.ac.kr (J.Y.); 2 Department of Automotive Electronics and Control Engineering, Hanyang University, Seoul 04763, Korea; luminar@hanyang.ac.kr

**Keywords:** connected and automated vehicle, cooperative perception, V2X communications, sensor fusion, advanced driver assistance system, trajectory prediction, risk assessment, collision warning

## Abstract

Driving environment perception for automated vehicles is typically achieved by the use of automotive remote sensors such as radars and cameras. A vehicular wireless communication system can be viewed as a new type of remote sensor that plays a central role in connected and automated vehicles (CAVs), which are capable of sharing information with each other and also with the surrounding infrastructure. In this paper, we present the design and implementation of driving environment perception based on the fusion of vehicular wireless communications and automotive remote sensors. A track-to-track fusion of high-level sensor data and vehicular wireless communication data was performed to accurately and reliably locate the remote target in the vehicle surroundings and predict the future trajectory. The proposed approach was implemented and evaluated in vehicle tests conducted at a proving ground. The experimental results demonstrate that using vehicular wireless communications in conjunction with the on-board sensors enables improved perception of the surrounding vehicle located at varying longitudinal and lateral distances. The results also indicate that vehicle future trajectory and potential crash involvement can be reliably predicted with the proposed system in different cut-in driving scenarios.

## 1. Introduction

Automated driving has generated increasing interest in recent decades due to its potential to address some of the most challenging issues faced by people and communities across the world. Road traffic crashes account for 1.35 million deaths a year worldwide, and they are the leading cause of death among children and young adults aged 5 to 29 years [1]. In addition, many people in different parts of the world still lack access to mobility, particularly in rural areas, and the number of older people who are no longer physically capable of driving has been increasing globally at the fastest rate recorded [2]. Another important issue in large metropolitan areas throughout the world is excessive traffic congestion, which is directly related with the growing number of vehicles on the road [2,3]. These challenges can be addressed by successful implementation of automated driving on public roads. With automated vehicles it is possible to achieve greater road safety, universal access to mobility, and higher transportation efficiency [4].

SAE International proposed different levels for driving automation, which include six levels ranging from no driving automation (level 0) to full driving automation (level 5) [5]. In level 0–2 systems, the driver is expected to respond to any evident vehicle system failure. A level 0 system does not provide any driving automation functions, while a level 1 system supports the driving task by performing either the longitudinal or the lateral motion control. A level 2 system performs both the longitudinal and the lateral motion control when engaged, and the driver is expected to supervise the automation system and take over the driving task whenever necessary to maintain safe driving. In level 3–5 systems, the driver determines whether to engage the automated driving system, and when engaged, the system performs the entire driving task. A level 3 system offers conditional driving automation, which permits the engagement of the automated driving functions only within its operational design domain (ODD). When engaged, the driver is expected to be responsive to a request to intervene and any system failures, and be ready to perform the driving task fallback in a timely manner. Similarly, a level 4 system also permits the automated driving engagement only within its ODD, but the driver is not expected to perform the driving task fallback and becomes a passenger of the vehicle while the system is engaged. Finally, a level 5 system permits the automated driving engagement under all driver-manageable on-road conditions, without limitations on the ODD.

Despite the considerable interest and effort devoted to automated driving in the past decades, most experts agree that level 5 systems are still decades away from becoming a reality on public roads [6]. At the moment, most automakers provide level 1 and 2 automation functions in the production vehicles. One of the most widely known level 2 systems is the Tesla Autopilot [7]. It has been stated that Tesla vehicles had logged a total of 3 billion miles with the Autopilot engaged as of February 2020 [8]. Tech companies such as Waymo and Uber have been working on the development of level 4 systems for ride-hailing services that involve a fleet of automated vehicles operating only within its ODD (e.g., specific geographical locations and appropriate weather conditions) [9]. The driving automation functions of automobiles require accurate and reliable perception of the surrounding environment, which is typically achieved by the use of remote sensors, such as radars, cameras, and lidars [10,11,12,13,14]. However, perception is a very challenging task due to the highly dynamic and complex nature of driving and traffic environment as well as varying lighting and weather conditions that affect the performance of the on-board sensors [15]. Perception errors in automated vehicles have resulted in a number of fatal crash incidents, and here we discuss some of the notable examples. In May 2016, the camera system of a Tesla Model S failed to distinguish the white side of a tractor trailer against the bright sky, and the vehicle hit the side of the trailer and passed underneath it, resulting in the first case of a traffic fatality involving automated vehicle technology [16]. In March 2018, the lane-keeping system of a Tesla Model X steered into a gore area and crashed into an impact attenuator when the perception system failed to recognize faded lane markings [17]. A crash incident that is very similar to the 2016 Tesla Model S crash occurred in March 2019, where a Tesla Model 3 crashed into the side of a tractor trailer and then drove beneath the trailer [18]. An Uber test vehicle was involved in a crash that resulted in a pedestrian fatality in March 2018, where the vehicle hit a pedestrian who was pushing a bicycle across the road at night [19]. The crash was predictable and avoidable, but a series of design flaws in the perception system contributed to the fatal outcome.

There has been increasing interest in connected and automated vehicles (CAVs) in recent years due to its potential to improve road safety, convenience, and energy efficiency [20,21,22]. The full benefits of automated driving can only be achieved when vehicles are capable of communicating and exchanging information with each other and also with the surrounding infrastructure. Vehicular wireless communications, which is often referred to as vehicle-to-everything (V2X) communications, incorporates various types of communication options depending on the participating entities. Some of the well-known types include vehicle-to-vehicle (V2V), vehicle-to-infrastructure (V2I), vehicle-to-pedestrian (V2P), and vehicle-to-network (V2N) communications. With V2X communications, it is possible to overcome the functional and environmental limitations of the on-board sensors. The advantages of V2X communications in terms of road safety and how it can complement and extend the perception methods based on automotive remote sensors have been described in more detail in [23].

The majority of recent studies on CAVs focus on cooperative adaptive cruise control, cooperative intersection control, and cooperative perception [22]. Although these studies present promising applications, not many studies have examined the benefits of the fusion of on-board remote sensors and cooperative approaches in the context of safety applications. An object association method based on V2V communications and on-board sensors was presented in [24], where the relative sender position and orientation were determined by using point matching algorithms. In [25], the plausibility checking of V2V communication data was implemented based on a multiple object tracking system with a camera sensor. Based on their evaluation, the authors reported that the proposed approach can overcome spoofing attacks if ghost vehicles are located within the camera field-of-view (FOV). A fusion approach based on radar and V2V communication data was suggested in [26], where an improved perception range and more accurate position and velocity estimates were obtained in a car-following scenario. In [27], an object matching algorithm based on V2V communication messages and radar measurements was presented and tested in highway driving scenarios, and the authors suggested that the track-to-track association algorithm can reliably handle the ambiguity issue in object matching. A simulation-based study on cooperative vehicle positioning was presented in [28], where average position errors were calculated for a varying percentage of the vehicles equipped with ranging sensors and V2V communication devices. A high-level fusion approach based on multiple on-board sensors and V2V/V2P communications was presented in [23] for the purpose of providing a timely warning prior to a possible collision. The proposed approach was evaluated in virtual driving environments, and the results demonstrated that reliable environment perception and collision prediction can be achieved by introducing V2X communications, even in scenarios where it is difficult to avoid a collision with existing safety systems based only on on-board sensors.

In this paper, we present the design and implementation of cooperative environment perception based on the fusion of V2X communications and automotive remote sensors. In continuation of our previous work [23], the Kalman-filter-based approach is employed for the high-level fusion of radar, camera, and V2X communication data, and the proposed cooperative approach is tested and evaluated in test-track environments. The experiments are carried out with two test vehicles, where each vehicle is equipped with a 5.9 GHz dedicated short-range communications (DSRC) transceiver along with a global navigation satellite system (GNSS) receiver for the exchange of vehicle state information. The host vehicle is additionally equipped with automotive radar and camera systems for remote sensing of the surrounding objects. The performance of the proposed approach for driving environment perception is evaluated at varying relative distances between the two test vehicles. The positioning accuracies at different longitudinal and lateral target vehicle positions are computed for each remote sensing system as well as for the proposed fusion method. The performance of the trajectory prediction and risk assessment is evaluated in different cut-in driving scenarios where the remote vehicle performs a lane-change maneuver in front of the host vehicle.

The rest of the paper is organized as follows. In Section 2, the overall architecture of the proposed driving environment perception system is described and background information about automotive remote sensors and V2X communications is provided. In Section 3, the proposed method for object state estimation and risk assessment is presented. The experimental results are reported and discussed in Section 4, and conclusions and directions for future research are presented in Section 5.

## 2. System Overview

### 2.1. Overall Design of the Proposed System

**Figure 1:**
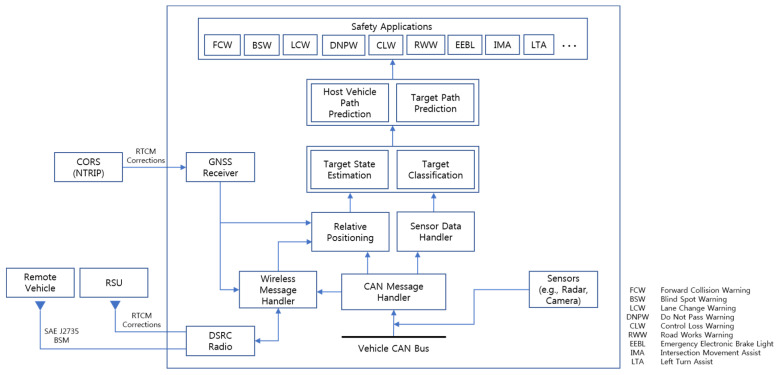
Functional blocks of the proposed cooperative environment perception system.

The overall design of the proposed cooperative environment perception approach based on on-board sensors and vehicular wireless communications is illustrated in Figure 1. The system is designed to serve as a flexible platform that enables reliable vehicle driving environment recognition and to provide appropriate road safety applications. Automotive remote sensors (e.g., radars and cameras) are often connected to the controller area network (CAN) bus such that the data from these sensors along with other data generated by in-vehicle electronic control units (ECUs) are collected in the form of CAN messages. The DSRC messages received with an on-board unit (OBU) are collected via Ethernet interfaces. For the enhancement of GNSS positioning performance, RTCM corrections, which contain differential corrections for the GNSS as defined by the Radio Technical Commission for Maritime Services (RTCM) Special Committee 104, can be obtained either through Networked Transport of RTCM via Internet Protocol (NTRIP) when an Internet connection is available, or through DSRC RTCM messages as defined in SAE J2735 [29] when it is possible to utilize roadside units (RSUs) capable of RTCM applications.

For cooperative relative positioning, and particularly for the fusion of the on-board sensors and V2X communications, it is necessary to transform the GNSS positioning information (i.e., latitude and longitude) of the host vehicle and the remote targets from the World Geodetic System 1984 (WGS 84) reference frame to the host vehicle reference frame. For this, WGS-84 coordinates are converted to earth-centered, earth-fixed (ECEF) coordinates and then to east-north-up (ENU) coordinates. The GNSS course heading of the host vehicle is utilized to finally rotate the remote target positions and find their relative positions in the host vehicle reference frame. The relative positions and other dynamic information on remote targets acquired from V2X communications can be used in conjunction with the measurements from on-board sensors for more accurate and reliable target state estimation, classification, and trajectory prediction. Some of the safety applications that can be offered with this cooperative approach are shown in the safety applications block in Figure 1.

### 2.2. Automotive Remote Sensors

The types of sensors used for driving environment perception and object tracking in recent years include radars, cameras, and lidars. These sensors perform detection of objects in the vehicle surroundings and provide information on the object state (e.g., relative position and relative speed) and object classification. Some of the most common advanced driver assistance system (ADAS) applications enabled by the environment perception technology include forward collision warning (FCW), automatic emergency braking (AEB), adaptive cruise control (ACC), lane departure warning (LDW), and lane keeping assist system (LKAS). For this study, we equipped our test vehicle with radar and camera sensors that have already been incorporated into production vehicles.

Automotive radar, which is an active ranging sensor designed for detecting and tracking remote targets in the surrounding environment, is one of the most used ranging sensors for ADAS functions these days. Automotive radars are capable of providing the relative position and speed information about the objects located within the sensor FOV, and they operate even in adverse weather conditions such as rain, fog, and snow. Two frequency bands, 24 GHz and 77 GHz, have been predominantly exploited for automotive radars [30,31,32]. The 24-GHz band is mainly used for short-range radar systems with a detection range up to 30 m [30,33], while the 77-GHz band is mainly used for long-range radar systems with a detection range up to 250 m [31]. A frequency modulated continuous wave (FMCW) radar is the most common type of automotive radars used for remote sensing of surroundings [31]. FMCW radars transmit a frequency-modulated signal in a continuous manner. The frequency of the signal changes linearly with time, and this enables accurate time measurement based on the frequency difference between the transmitted and received signals, which can be converted into range information. The specification of Delphi ESR 2.5, the multimode radar system utilized for the driving experiments in this study, is shown in Table 1.

Computer vision techniques are increasingly utilized for automotive safety applications. Camera systems with computer vision methods perform detection and localization of the objects that have been captured in the camera images. It is also possible to extract valuable information on the driving environment, such as lane marking positions and road curvature, from the images obtained with the cameras on board the vehicle. A comprehensive review on computer vision techniques for vehicle detection, tracking, and behavior characterization is presented in [34]. The most notable camera system that has been widely incorporated into production vehicles is that of Mobileye [35]. The longitudinal distance to the surrounding vehicle can be determined based on the image position of the target and the detected target width or height [36,37,38]. Based on [36], the accuracy of the range estimated with the camera system decreases quadratically as the distance to the object increases, while the error percentage increases linearly, such that a 5% error in range is expected at 45 m and a 10% error in range is expected at 90 m. The characteristics of the Mobileye 630 camera system that was installed on our test vehicle are summarized in Table 2.

Lidar stands for light detection and ranging, and as the name suggests, it measures the relative distance and angle to a target within its FOV by transmitting laser light and measuring reflected light. Near-infrared light with a wavelength of 905 nm is typically used for automotive lidars, and they are capable of higher localization performance compared with other automotive ranging sensors. Lidar sensors attracted much attention of the automated driving research community since they were used by many groups who participated in the DARPA Grand Challenges [39,40,41]. Despite the advantage of lidar sensors, most automakers are yet to incorporate them into production vehicles largely due to their drawbacks such as the high manufacturing cost and bulky form factor. Moreover, high-performance lidars generate millions of data points per second [42]. Processing lidar data points is often computationally expensive and the perception and tracking performance based on lidar measurements can vary depending on the algorithm used [43,44,45]. The use of lidar sensors is outside the scope of this paper, and perception approaches based on multiple remote sensors including lidars will be investigated in future work.

### 2.3. V2X Communications

There exists a range of wireless communication technologies that can be employed for CAV systems and intelligent transportation system (ITS) applications (e.g., 5.9 GHz DSRC, cellular communications, Wi-Fi, Bluetooth, satellite radio, and visible light communications) [20,46]. The V2X communications based on 5.9 GHz DSRC is a mature technology that is the most widely tested and commercially available [46,47,48]. The DSRC-based V2X communication technology is based on the IEEE 802.11p and the IEEE 1609 series of standards, which are collectively known as wireless access in vehicular environments (WAVE) standards [46]. The 5.9 GHz frequency band is divided into seven 10-MHz channels, which include one control channel (CCH) and six service channels (SSHs) [49]. As an alternative to the DSRC-based V2X communications, the cellular V2X (C-V2X) communications based on the 3GPP standards has attracted significant attention in recent years. The C-V2X communications utilizes the cellular network infrastructure (e.g., LTE and 5G networks) to enable V2X applications. For the upper layers, the C-V2X technology is expected to leverage existing standards (e.g., IEEE, SAE, ISO, and ETSI) and utilize common ITS message types, such as the basic safety message (BSM) of IEEE WAVE and the cooperative awareness message (CAM) and the decentralized environmental notification message (DENM) of ETSI ITS-G5. Some of the advantages of the C-V2X technology over 5.9 GHz DSRC include a much larger coverage area, higher throughput, lower latency, and more robust scalability [47,48]. Despite the advantages, the standardization work of C-V2X communications is still in process and the availability of C-V2X commercial hardware equipment is limited at this stage. In this work, we utilized 5.9 GHz DSRC for V2X communications, and the test vehicles were equipped with Cohda MK5 OBUs for the exchange of information among vehicles. The characteristics of DSRC-based V2X communications are shown in Table 3.

The vehicle state information is broadcast and shared among the vehicles equipped with DSRC devices by exchanging the BSM, which is defined in the SAE J2735 message set dictionary [29]. The BSM contains data obtained from the vehicle CAN bus and the GNSS receiver, which include safety critical state information such as the vehicle position, heading, speed, and yaw rate. The BSM is typically transmitted with a period of 100 ms on the dedicated safety channel (i.e., Channel 172 in the U.S.) [29,46]. The BSM Part I data contain the BSM core data frame that shall be included when broadcasting a BSM, while the BSM Part II data contain optional additional information (e.g., event flags, path history, path prediction, and exterior lights). The contents of the BSM core data frame are described in Table 4.

## 3. State Estimation and Prediction

A Kalman-filter-based fusion approach that was previously described in [23] is employed in this study for state estimation and trajectory prediction of the remote targets in the vehicle surroundings. At each time step, the measurements from the on-board remote sensors as well as the BSMs received from the remote targets are collected and processed with Kalman filter algorithms, which reduce the measurement noise and output the state and error covariance for each track. The types of information we used in this study to estimate the state and future trajectory of the remote target include the following: Position, speed, heading, yaw rate, and size information from the V2X communications; range and azimuth information from the radar system; and longitudinal and lateral distance information from the camera system. The state estimates from different tracks are then associated and fused together, where the weight for each track is determined based on the error covariance. The future trajectory of the remote target detected in the perception stage is estimated with the constant turn rate and velocity (CTRV) motion model. For performance evaluation in the context of safety applications, the future trajectory of the remote target is compared with the future trajectory of the host vehicle and an appropriate warning is generated when a possible collision is detected.

### 3.1. Kalman Filtering

**Figure 2:**
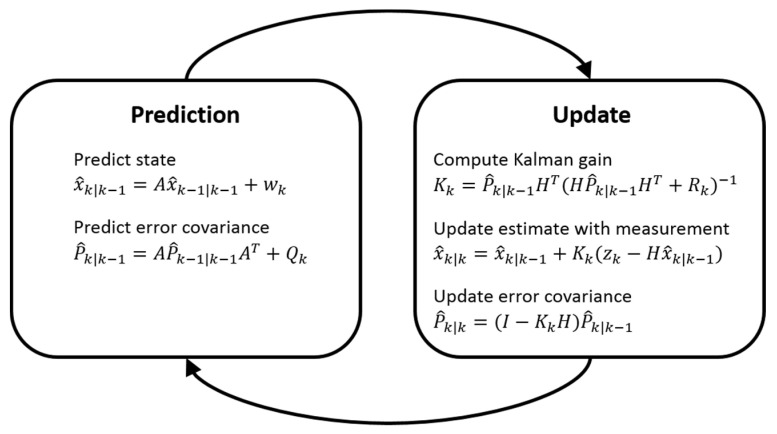
Operation of the Kalman filter algorithm.

Kalman filtering [50,51] is a recursive algorithm that estimates the state of a system as well as the estimation uncertainty based on the prior state and the noisy measurements. The operation of the Kalman filter is described in Figure 2. In the prediction step, the state ${\hat{x}}_{k|k-1}$ and the error covariance ${\hat{P}}_{k|k-1}$ are projected with the state transition matrix A from the previous state ${\hat{x}}_{k-1|k-1}$ and the corresponding error covariance ${\hat{P}}_{k-1|k-1}$. The random variable $w_{k}$ is the process noise, which is assumed to be normally distributed with the process noise covariance $Q_{k}$, such that $w_{k}\;\sim \;N\left(0,Q_{k}\right)$. The process noise covariance $Q_{k}$ assumed here to be constant, but it may be changed during the filter operation for adjustment to different dynamics. In the update step, the error covariance ${\hat{P}}_{k|k-1}$ is used along with the measurement matrix H and the measurement error covariance $R_{k}$ to compute the Kalman gain $K_{k}$. The measurement matrix H maps the state vector $x_{k}$ to the measurement vector $z_{k}$ such that

(1) $$z_{k}=Hx_{k}+v_{k}.$$

The random variable $v_{k}$ is the measurement noise, which is assumed to be normally distributed with the measurement error covariance $R_{k}$, such that $v_{k}\;\sim \;N\left(0,R_{k}\right)$. Finally, the state ${\hat{x}}_{k|k}$ and the error covariance ${\hat{P}}_{k|k}$ are updated based on the Kalman gain $K_{k}$ and the measurement vector $z_{k}$ obtained at time step k.

The relative positions and the motion equations of the remote targets in the vehicle surroundings are typically given in Cartesian coordinates, and it is necessary to perform a polar-to-Cartesian transformation when the ranging measurements obtained from remote sensors (e.g., radar sensors) are in polar coordinates. An extended Kalman filter (EKF) is often utilized to handle such nonlinear systems; however, the linear approximation of a nonlinear system may result in highly unstable performance, and the derivation of the Jacobian matrices is often nontrivial in many applications [52]. More advanced nonlinear filtering approaches such as sequential Monte Carlo methods, also known as particle filters, have been introduced [53], but the computation complexity becomes enormous for high dimensional problems and the use of particle filters should be determined based on the degree of the system nonlinearity [54]. In this study, the unbiased converted measurement Kalman filter algorithm as presented in [55,56] is employed to perform the coordinate transformation without bias and to obtain the correct covariance. This converted measurement approach yields nearly optimal estimates and provides higher estimation accuracy than the EKF [57].

**Figure 3:**
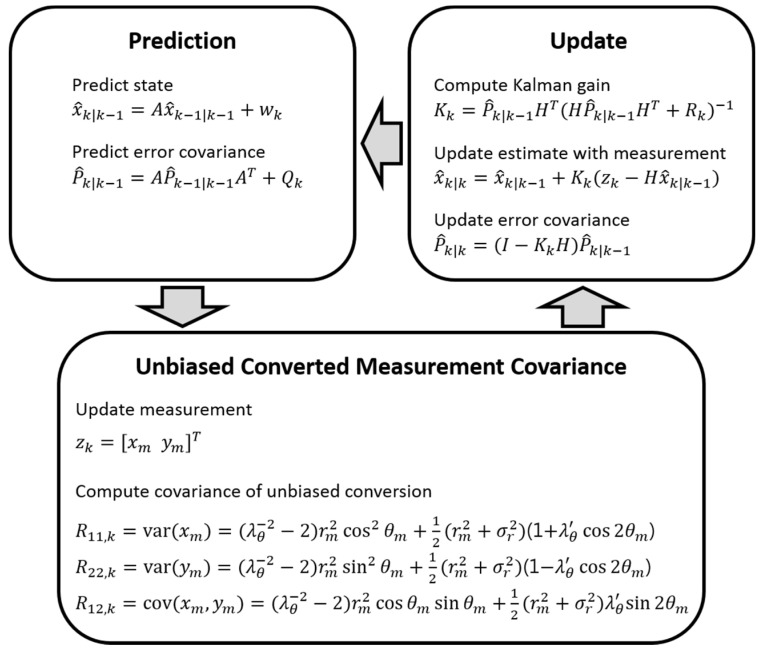
Operation of the unbiased converted measurement Kalman filter algorithm.

The operation of the unbiased converted measurement Kalman filter is described in Figure 3. The filtering process includes additional steps to compute the covariance of the unbiased conversion compared with the linear Kalman filtering described above. The range measurement $r_{m}$ and the azimuth measurement $\theta_{m}$ are defined as

(2) $$r_{m}=r+v_{r}$$

(3) $$\theta_{m}=\theta +v_{\theta }$$

where r and θ are the true range and azimuth of the remote target, respectively, and $v_{r}$ and $v_{\theta }$ are the associated measurement noise with an error standard deviation of $\sigma_{r}$ and $\sigma_{\theta }$, respectively. The unbiased converted measurements $x_{m}$ and $y_{m}$ are computed by taking into account the bias compensation factor such that

(4) $$x_{m}=\lambda_{\theta }^{-1}r_{m}\cos\theta_{m}$$

(5) $$y_{m}=\lambda_{\theta }^{-1}r_{m}\sin\theta_{m}.$$

The computation of the unbiased converted measurements as well as the associated covariance requires the compensation factors $\lambda_{\theta }$ and $\lambda_{\theta }^{\prime }$, which are determined from

(6) $$\lambda_{\theta }=E\left[\cos v_{\theta }\right]=e^{-\sigma_{\theta }^{2}/2}$$

(7) $$\lambda_{\theta }^{\prime }=E\left[\cos2v_{\theta }\right]=e^{-2\sigma_{\theta }^{2}}.$$

The rest of the steps for obtaining the covariance of the unbiased conversion are as shown in Figure 3, and the state ${\hat{x}}_{k|k}$ and the error covariance ${\hat{P}}_{k|k}$ are updated according to the Kalman gain $K_{k}$ and the measurement $z_{k}$.

### 3.2. Data Fusion

**Figure 4:**
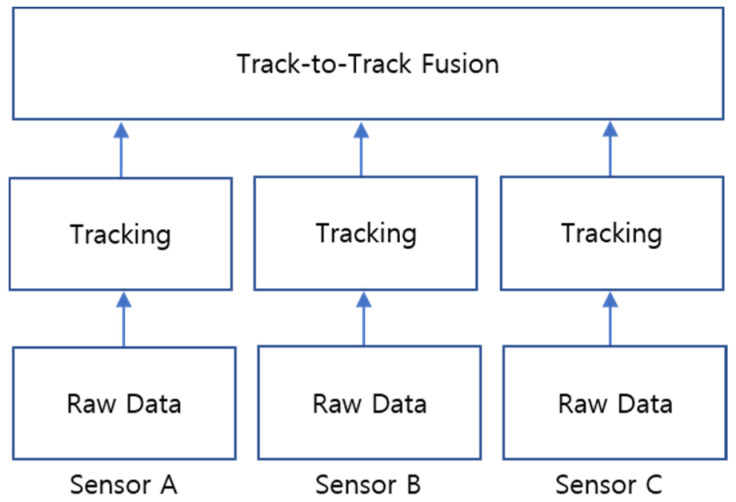
High-level fusion system architecture.

A track-to-track fusion approach is employed in this study for combining high-level data from multiple sources. The data processing for each sensor system is performed individually at the sensor level in a high-level fusion system. Each sensor system outputs one or more tracks based on the sensor measurements, and the state estimates from multiple sensor tracks are associated and combined with a track-to-track fusion algorithm. A high-level fusion of multiple sensors has been successfully implemented in many studies dealing with automotive applications [58,59,60,61,62]. Important advantages of the high-level fusion approach lie in spatial and temporal alignment, modularity, and communication overhead [63,64]. A simple block diagram for the high-level fusion system architecture [63,65] is shown in Figure 4.

One of the most widely used algorithms for track-to-track fusion is the convex combination algorithm [65,66,67,68], and it has been used extensively for its simple implementation. Two state estimates $x_{i}$ and $x_{j}$ with the corresponding covariance $P_{i}$ and $P_{j}$, respectively, can be fused to obtain the state estimate $\overset{ˇ}{x}$ by

(8) $$\overset{ˇ}{x}=P_{j}{\left(P_{i}+P_{j}\right)}^{-1}x_{i}+P_{i}{\left(P_{i}+P_{j}\right)}^{-1}x_{j}\;=\overset{ˇ}{P}\left(P_{i}{}^{-1}x_{i}+P_{j}{}^{-1}x_{j}\right)$$

where $\overset{ˇ}{P}$ is the covariance associated with the fused estimate, which is given by

(9) $$\overset{ˇ}{P}=P_{i}-P_{i}{\left(P_{i}+P_{j}\right)}^{-1}P_{i}\;=\;P_{i}{\left(P_{i}+P_{j}\right)}^{-1}P_{j}\;=\;{\left(P_{i}{}^{-1}+P_{j}{}^{-1}\right)}^{-1}.$$

### 3.3. Trajectory Prediction and Risk Assessment

The future trajectory of the remote target in the vehicle surroundings is estimated with a CTRV model. The state space at time step k is defined as

(10) $$x_{k}={\left[X_{k}\;Y_{k}\;v_{k}\;\psi_{k}\;\omega_{k}\right]}^{T}$$

where $X_{k}$ is the relative distance in the longitudinal direction, $Y_{k}$ is the relative distance in the lateral direction, $v_{k}$ is the target speed, $\psi_{k}$ is the relative course heading, and $\omega_{k}$ is the yaw rate. The state transition equation for the prediction of the state at time step k+1 is given by

(11) $$x_{k+1}=x_{k}+\left[\begin{array}{c} \begin{array}{c} \frac{v_{k}}{\omega_{k}}\left(\sin\left(\psi_{k}+\omega_{k}\Delta t\right)-\sin\left(\psi_{k}\right)\right)\\ \;\frac{v_{k}}{\omega_{k}}(-\cos\left(\psi_{k}+\omega_{k}\Delta t\right)+\cos\left(\psi_{k}\right))\\ 0 \end{array}\\ \omega_{k}\Delta t\\ 0 \end{array}\;\right]\;.$$

**Figure 5:**
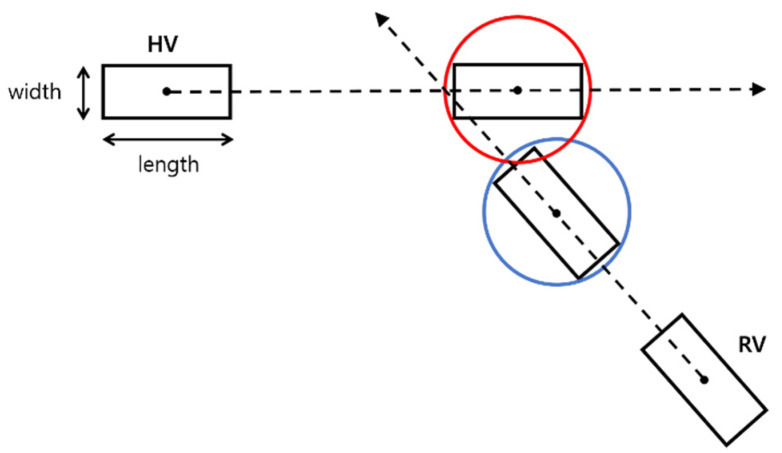
Illustration of collision prediction based on the circle model [23].

For the purpose of risk assessment, the circle model described in [23] is employed and a possible collision event is predicted based on the future trajectories of the remote vehicle and the host vehicle as shown in Figure 5. The host vehicle radius $R_{HV}$ and the remote vehicle radius $R_{RV}$ are given by

(12) $$R_{HV}=\frac{\sqrt{W_{HV}{}^{2}+L_{HV}{}^{2}}}{2}$$

(13) $$R_{RV}=\frac{\sqrt{W_{RV}{}^{2}+L_{RV}{}^{2}}}{2}$$

where $W_{HV}$ and $L_{HV}$ denote the width and the length of the host vehicle, respectively, and $W_{RV}$ and $L_{RV}$ denote the width and the length of the remote vehicle, respectively. A possible collision is determined if the following inequality is true:

(14) $$\sqrt{{\left(X_{HV}-X_{RV}\right)}^{2}+{\left(Y_{HV}-Y_{RV}\right)}^{2}}\leq R_{HV}+R_{RV}\;.$$

The detection of a possible collision leads to the generation of a collision warning to the host vehicle. Based on the time-to-collision (TTC) estimate, four different collision warning messages are provided. Following the collision warning stages discussed in [69], the warning messages provided by the proposed system include “no threat” for no possible collision, “threat detected” for TTC>2.6 s, “inform driver” for 1.6 s<TTC≤2.6 s, and “warn driver” for TTC≤1.6 s. The description for each warning message is described in Table 5. The conditions for the collision warning messages defined here are similar to those of Daimler PRE-SAFE [70] and Mobileye FCW [71]. The PRE-SAFE and Mobileye systems warn the driver approximately 2.6 s and 2.7 s before the expected collision, respectively. In the case of the PRE-SAFE system, an additional warning is provided at approximately 1.6 s before the expected collision.

## 4. Experimental Evaluation

The proposed system for cooperative driving environment perception was evaluated experimentally in test-track environments. Two test vehicles were utilized for the experiments where both vehicles were equipped with V2X communication devices such that the exchange of the BSM between the two test vehicles was enabled. In order to evaluate the benefits of introducing V2X communications to the currently available environment perception systems, the host vehicle in this work was additionally equipped with radar and camera systems that have already been adopted in production vehicles.

### 4.1. Vehicle Configuration

**Figure 6:**
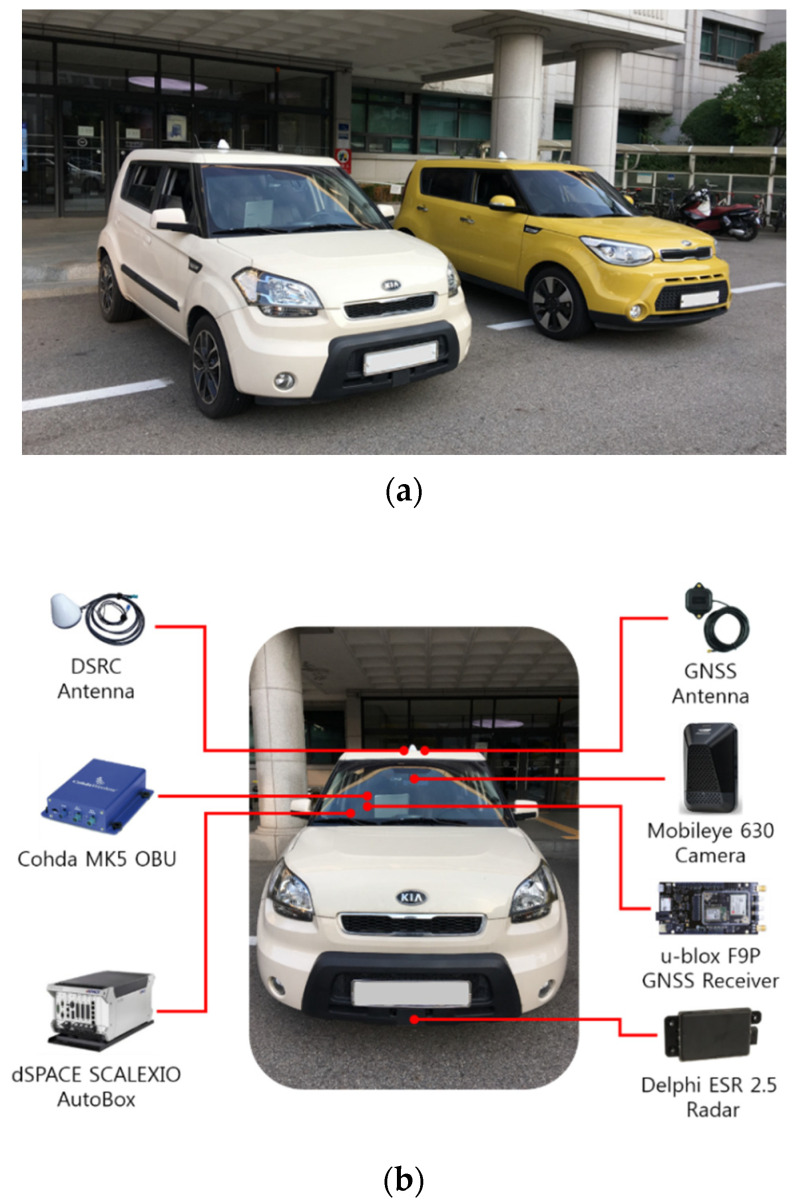
Test vehicles used for the driving experiments. (**a**) The host vehicle (white) and the remote vehicle (yellow); (**b**) experimental equipment installed on the test vehicle.

Two Kia Soul cars, one in white and the other in yellow as shown in Figure 6a, were used for the experiments. The experiment setup consisted of a combination of hardware and software components that enabled the host vehicle to gather information on the vehicle surroundings by means of on-board remote sensors and V2X communications. As shown in Figure 6b, the host vehicle (Kia Soul in white) was equipped with a Delphi ESR 2.5 radar and a Mobileye 630 camera for detection of surrounding objects, and each test vehicle was equipped with a Cohda MK5 OBU, a DSRC antenna, a u-blox F9P GNSS receiver, and a GNSS antenna, all of which together enabled the vehicles to exchange BSM via V2X communications and share vehicle position information with a lane-level accuracy. The u-blox F9P device is a low-cost GNSS module with built-in support for standard RTCM corrections.

**Figure 7:**
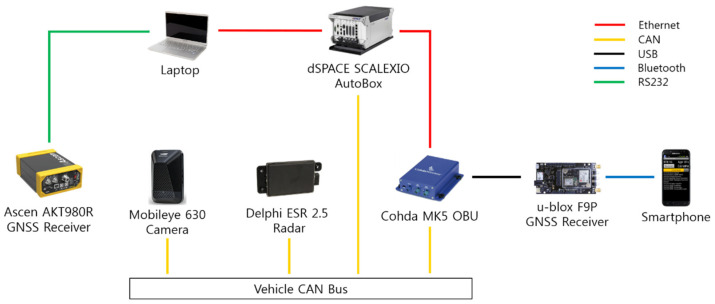
Overview of the hardware interface.

**Figure 8:**
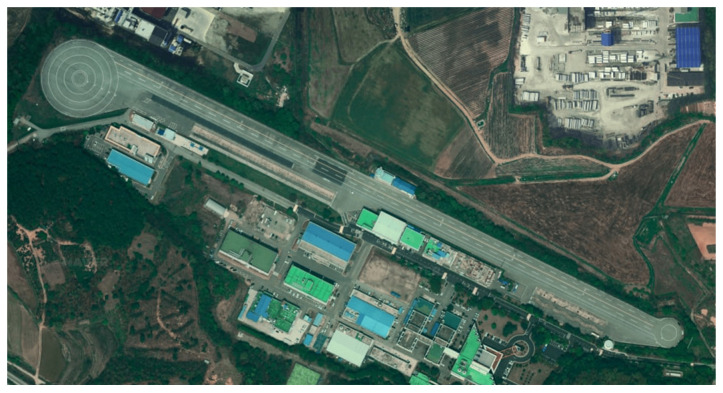
Proving ground at the Korea Automotive Technology Institute.

For real-time data acquisition and processing, the host vehicle was equipped with a dSPACE SCALEXIO AutoBox, which is an in-vehicle prototyping system with a real-time processor and interfaces for CAN and Ethernet communications. The signals transmitted from the radar and camera systems as well as the in-vehicle ECUs were acquired via CAN interfaces, and the signals transmitted from the DSRC OBU were acquired via Ethernet interfaces. RTCM corrections were acquired with an NTRIP client running on a smartphone and streamed via a Bluetooth connection to the u-blox C099-F9P application board. In order to obtain ground truth positions for the test vehicles, both vehicles were equipped with an Ascen AKT980R, which is a highly accurate GNSS receiver that provides GNSS carrier phase measurements with a horizontal positioning accuracy of 0.008 m and a heading accuracy of 0.09 deg with a 2-m baseline. The overview of the hardware interface for the proposed system is presented in Figure 7. The driving experiments were conducted at the proving ground available at Korea Automotive Technology Institute which is shown in Figure 8.

### 4.2. Surrounding Vehicle Localization

#### 4.2.1. Experimental Environment

**Figure 9:**
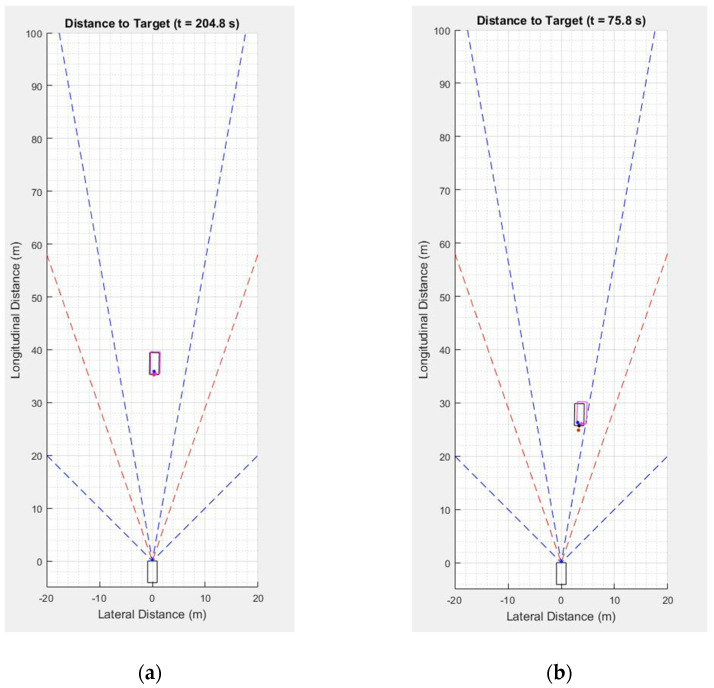
Normal driving scenarios considered for the localization performance evaluation of the proposed method. (**a**) Both vehicles driving in the same lane; (**b**) remote vehicle driving in the adjacent lane.

In order to obtain relative position estimates of the remote vehicle from the radar and camera and also from V2X communications, driving experiments were conducted under the scenarios where the remote vehicle is driven ahead the host vehicle. For the performance evaluation of the proposed data fusion approach for different relative target locations, the relative distance between the two test vehicles was gradually increased. As shown in Figure 9, the first set of experiments was carried out under a scenario where both test vehicles drove in the same lane, and the second set of experiments was conducted under a scenario where the remote vehicle drove in the adjacent lane. The two driving scenarios, which are referred to as the “normal driving scenarios” here, are summarized in Table 6. For these two normal driving scenarios, dynamic driving maneuvers such as lane change maneuvers were not performed. For both driving scenarios, the host vehicle was driven at a speed of about 20 km/h while the remote vehicle was driven at about 25 km/h. The red and blue dashed lines in Figure 9 indicate the FOV of the camera and radar sensors, respectively. The narrower set of dashed lines in blue indicates the long-range radar coverage, whereas the wider set indicates the mid-range radar coverage. The relative positions of the target vehicle obtained from the radar, camera, and V2X communications are plotted in different colors. The red dot indicates the camera measurement while the blue dot indicates the radar measurement. The dot in magenta indicates the relative position obtained based on V2X communication data, and finally black dot denotes the ground truth position. All of these dots represent the estimated distance to the rear center of the remote vehicle from the origin, which is the center of the front bumper of the host vehicle. The magenta bounding box is created and rotated based on the width, length, and heading information obtained from V2X communications at a given time step. Finally, the black bounding box represents the ground truth position of the remote vehicle.

#### 4.2.2. Performance Evaluation

**Figure 10:**
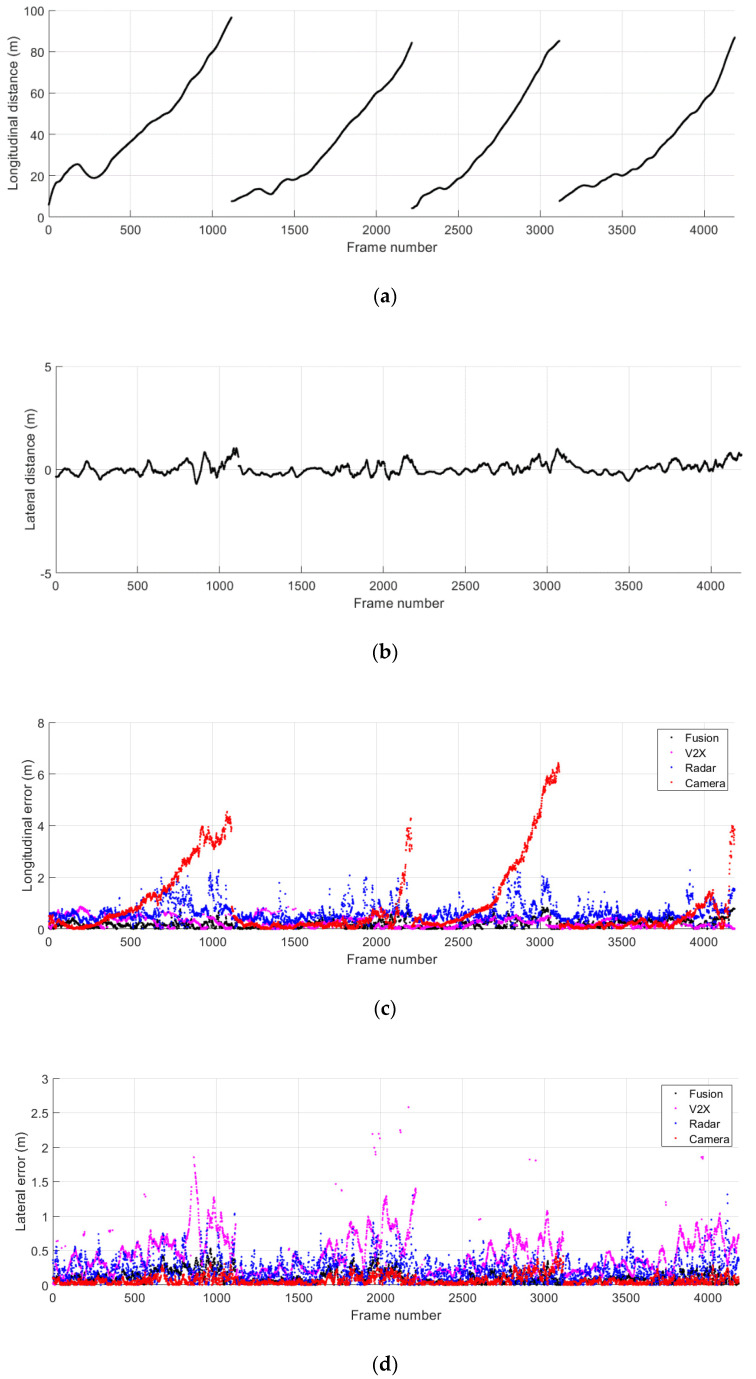
Relative distance to the remote vehicle during the normal driving scenario 1. (**a**) Ground truth longitudinal distance; (**b**) ground truth lateral distance; (**c**) absolute error for longitudinal distance estimates; (**d**) absolute error for lateral distance estimates.

Figure 10a,b show the ground truth distance to the remote vehicle obtained for the driving scenario where both vehicles are in the same lane. The longitudinal and lateral relative distance obtained from separate test runs are concatenated and presented together. The time step between two consecutive frames from the same test run corresponds to 0.1 s. The separation between the two test vehicles gradually increases while both vehicles stay in the same lane. The absolute error for the relative position in the longitudinal and lateral directions is presented in Figure 10c,d. The results of the proposed data fusion method are presented along with those of individual remote sensing systems. Despite the fluctuations observed in the position estimates of the radar, camera, and V2X communications, the proposed method enables reliable estimation of the relative position of the remote target at varying distances. 

As presented in Table 7 and Table 8, the performance of the proposed data fusion method is evaluated by computing the root mean squared error (RMSE) and the standard deviation of the error, and compared with those calculated for other remote sensing systems. In order to determine the positioning accuracy at varying relative distances, the results are grouped in separate 10-m bins (in the longitudinal direction). For the driving scenario with both vehicles in the same lane, the longitudinal and lateral localization accuracies of the proposed method in terms of the RMSE are found to be 0.22 m and 0.13 m, respectively, when taking into account all the results that correspond to the range of longitudinal distances between 0 and 70 m. 

The results obtained when the target vehicle is at a relative longitudinal distance of 70 m or longer are not used for this performance evaluation, considering that the lateral position accuracy of the GNSS system used for ground truth degrades at such long distances due to the limited heading accuracy such that it may not be appropriate to be utilized as a reference system. In addition, the Mobileye camera used in this work appears to suffer from significant degradation in longitudinal accuracy for remote targets located at such distances.

**Figure 11:**
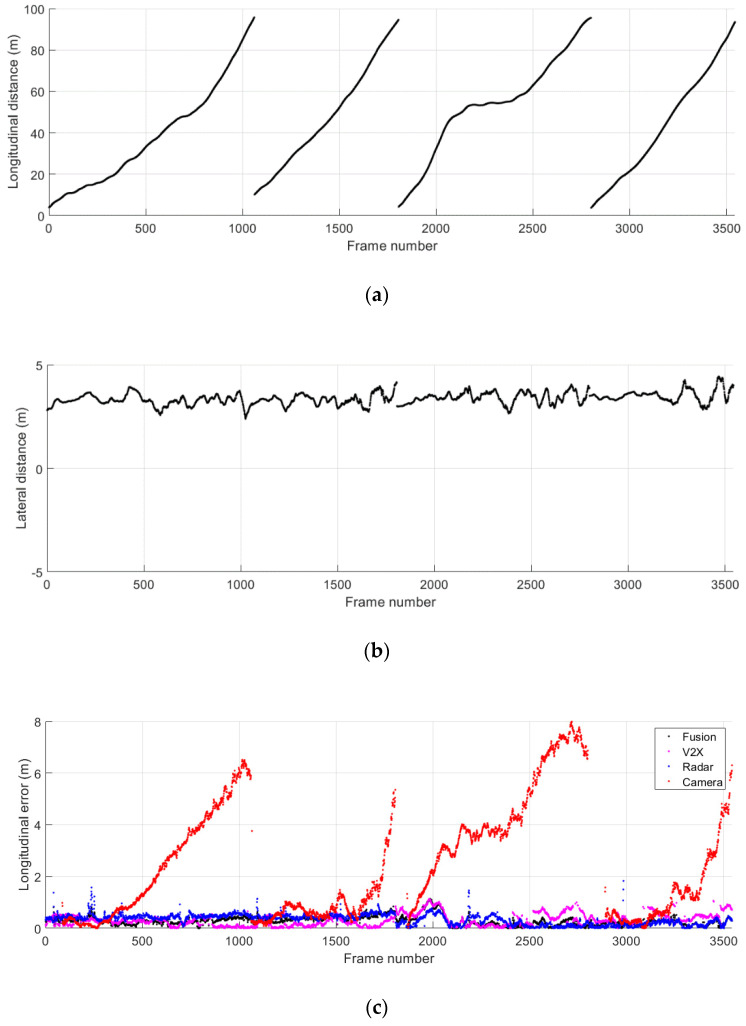

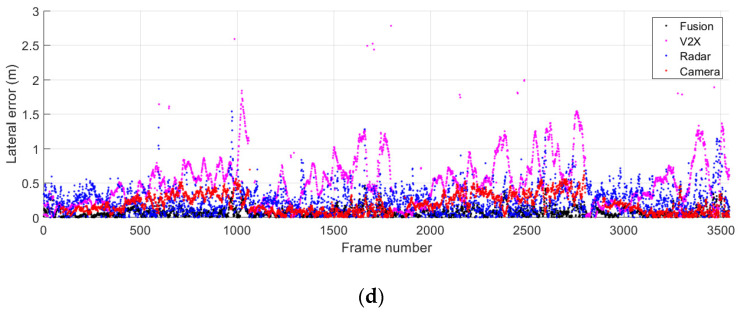
Relative distance to the remote vehicle during the normal driving scenario 2. (**a**) Ground truth longitudinal distance; (**b**) ground truth lateral distance; (**c**) absolute error for longitudinal distance estimates; (**d**) absolute error for lateral distance estimates.

The ground truth distance to the remote vehicle during the driving scenario where the remote vehicle is in the adjacent lane is shown in Figure 11a,b. As previously explained, the relative positions of the remote vehicle acquired from separate test runs are concatenated and presented together, and the time step between two consecutive frames from the same test run corresponds to 0.1 s. The separation between the two test vehicles gradually increases while the remote vehicle stays in the adjacent lane. The absolute error for the longitudinal and lateral distances to the remote vehicle is presented in Figure 11c,d. Similar to the aforementioned case with both vehicles in the same lane, the proposed fusion method reliably estimates the remote target position at varying distances even at times when the accuracy of individual remote sensors becomes severely degraded.

The performance of the individual remote sensing systems and the proposed cooperative environment perception approach is evaluated by computing the RMSE and the standard deviation of the error at different relative distances as shown in Table 9 and Table 10. Similar to the previous scenario, the results are grouped in separate 10-m bins (in the longitudinal direction) to determine the positioning accuracy at varying relative distances for the driving scenario with the remote vehicle driving in the adjacent lane. Taking into account all of the position estimates that fall between 0 and 70 m in the longitudinal direction, the longitudinal and lateral localization accuracies of the proposed method in terms of the RMSE are found to be 0.33 m and 0.09 m, respectively.

**Figure 12:**
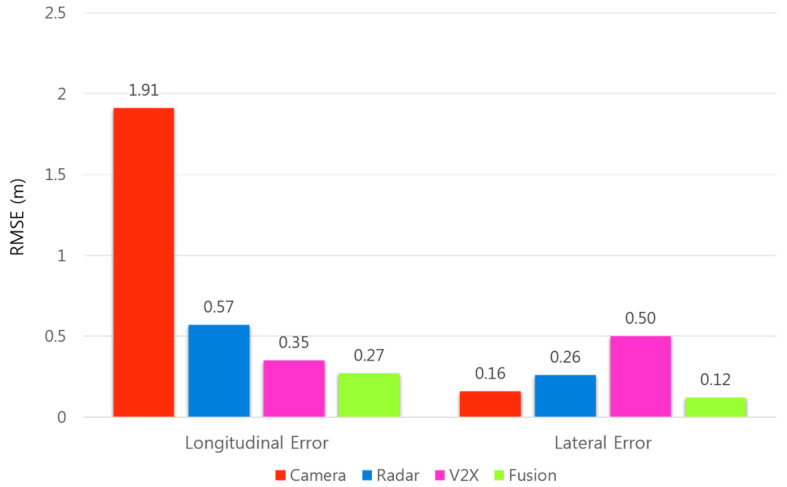
Relative positioning accuracy in the longitudinal and lateral directions for the combined sets of the measurements obtained from the two normal driving scenarios.

Figure 12 shows the relative positioning accuracy in the longitudinal and lateral directions for the combined sets of the measurements from the two normal driving scenarios. In the longitudinal direction, the total RMSE of the relative position estimated with the proposed fusion approach is 0.27 m, which corresponds to an improvement of 86% and 52% compared to the total RMSE of the relative position estimated with the camera and radar sensors, respectively. In the lateral direction, the total RMSE of the relative position estimated with the proposed fusion approach is 0.12 m, which corresponds to an improvement of 27% and 56% compared to the total RMSE of the relative position estimated with the camera and radar sensors, respectively.

### 4.3. Cut-In Driving Scenario

#### 4.3.1. Experimental Environment

The performance of the proposed cooperative environment perception approach was also evaluated in cut-in driving scenarios where the remote vehicle performs a lane-change maneuver in front of the host vehicle. Three different cut-in scenarios were considered in this work as described in Table 11. The host vehicle traveled at 40–45 km/h at the start of the cut-in maneuver in all three cut-in driving scenarios, while the remote vehicle originally driving in the adjacent lane cut in at a distance of 15–20 m in front of the host vehicle. The speed of the remote vehicle was set differently for each cut-in scenario in order to vary the level of collision threat, such that the scenario 1 presents the lowest level of threat while the highest level of threat is expected in the scenario 3.

#### 4.3.2. Performance Evaluation

**Figure 13:**
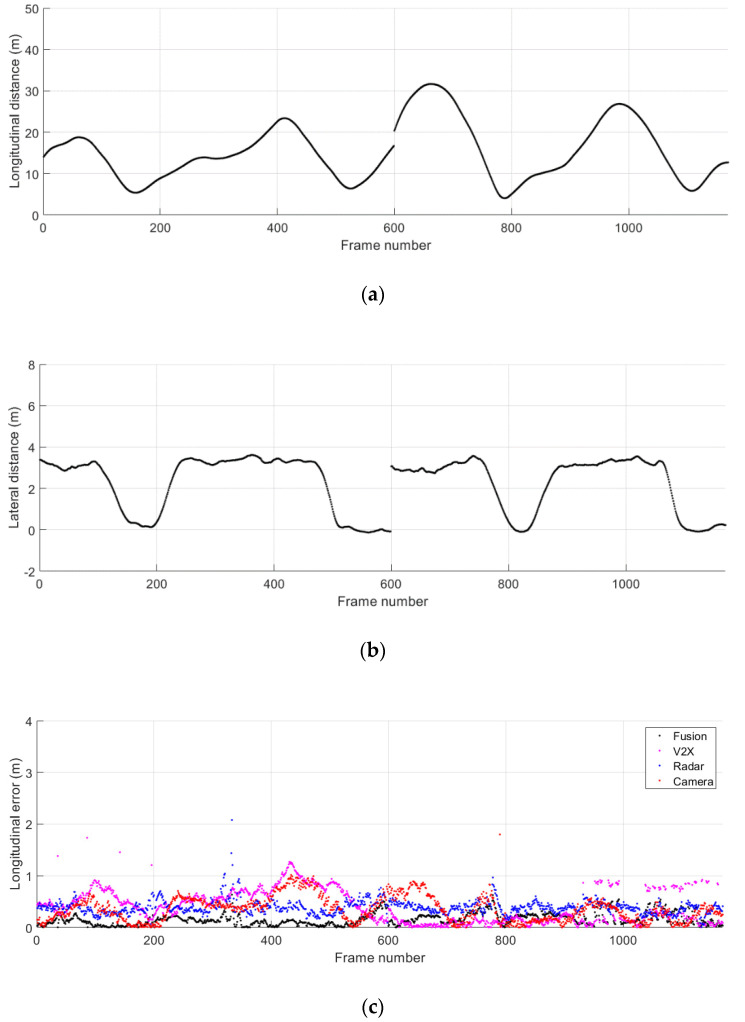

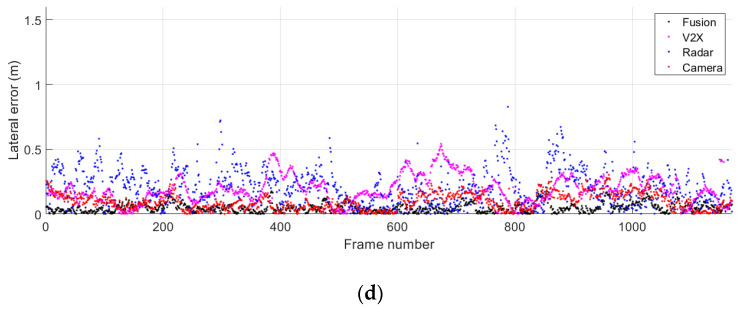
Relative distance to the remote vehicle during the cut-in driving scenario 1. (**a**) Ground truth longitudinal distance; (**b**) ground truth lateral distance; (**c**) absolute error for longitudinal distance estimates; (**d**) absolute error for lateral distance estimates.

**Figure 14:**
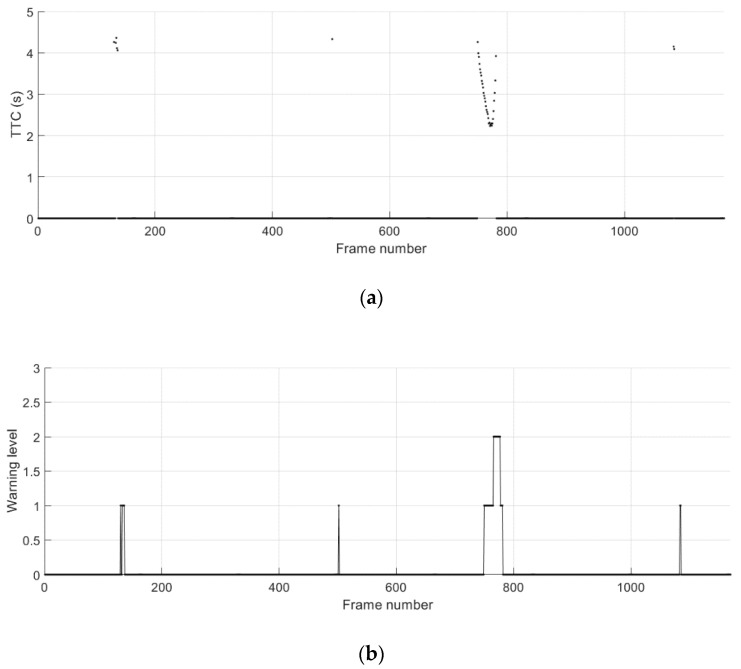
Potential crash events predicted during the cut-in driving scenario 1. (**a**) Time-to-collision (TTC); (**b**) collision warning level.

The ground truth relative position of the remote vehicle and the absolute error for the relative position during the cut-in driving scenario 1 are shown in Figure 13. The longitudinal and lateral relative distance measurements obtained from separate test runs are concatenated and shown together. The start and the duration of the cut-in events can be conveniently observed in Figure 13b, where the lateral distance changes from the center of the adjacent lane towards the center of the lane that the host vehicle is positioned. The TTC results and the corresponding levels of the collision warning obtained from the proposed method for trajectory prediction and risk assessment are presented in Figure 14. Possible collision events are successfully predicted for all four cut-in maneuvers performed by the remote vehicle. The TTC results for the collision detection shown are above 4 s except the third cut-in case. The levels of these threats other than the third one are minor, resulting in level-1 warnings only for a short duration of time. A level-2 warning is given for the third cut-in event, which is due to the more abrupt change in the longitudinal distance just prior to and during the lane-change maneuver.

**Figure 15:**
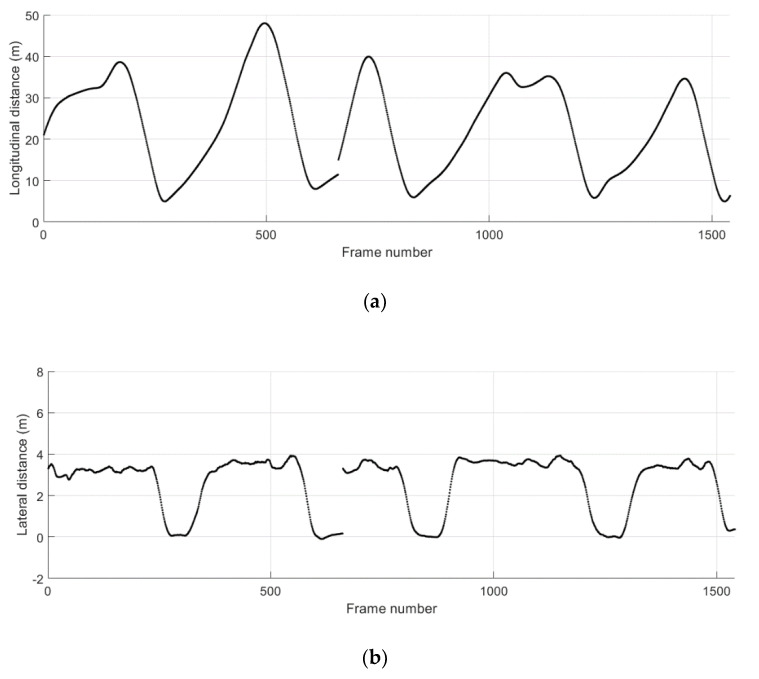

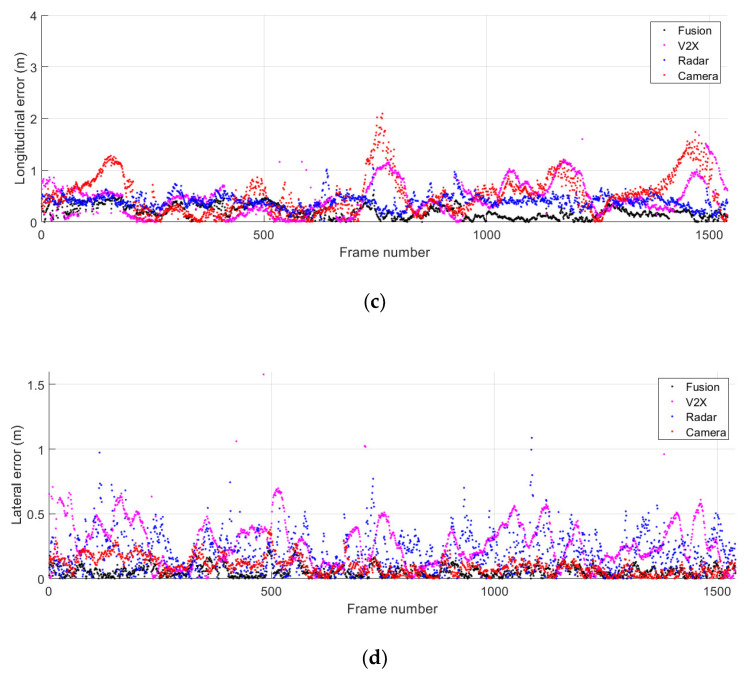
Relative distance to the remote vehicle during the cut-in driving scenario 2. (**a**) Ground truth longitudinal distance; (**b**) ground truth lateral distance; (**c**) absolute error for longitudinal distance estimates; (**d**) absolute error for lateral distance estimates.

**Figure 16:**
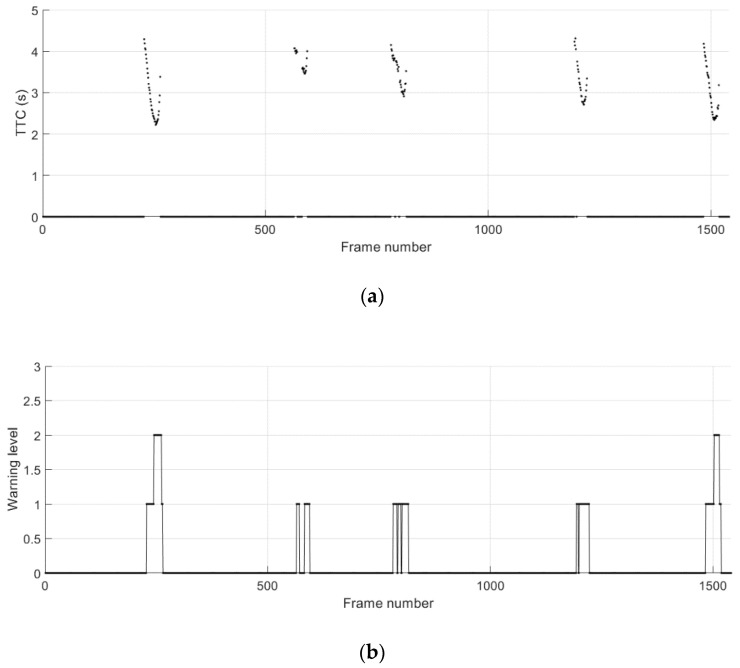
Potential crash events predicted during the cut-in driving scenario 2. (**a**) Time-to-collision (TTC); (**b**) collision warning level.

Figure 15 shows the ground truth position and the absolute position error in longitudinal and lateral directions during the cut-in driving scenario 2. The data collected from separate test runs are concatenated as presented. A total of five cut-in events take place in this scenario, and the start and the duration of these events can be observed in Figure 15b. The TTC results and the corresponding collision warning levels are presented in Figure 16. For all five cut-in maneuvers performed by the remote vehicle in the scenario 2, possible collision events are correctly predicted and corresponding collision warnings are provided.

**Figure 17:**
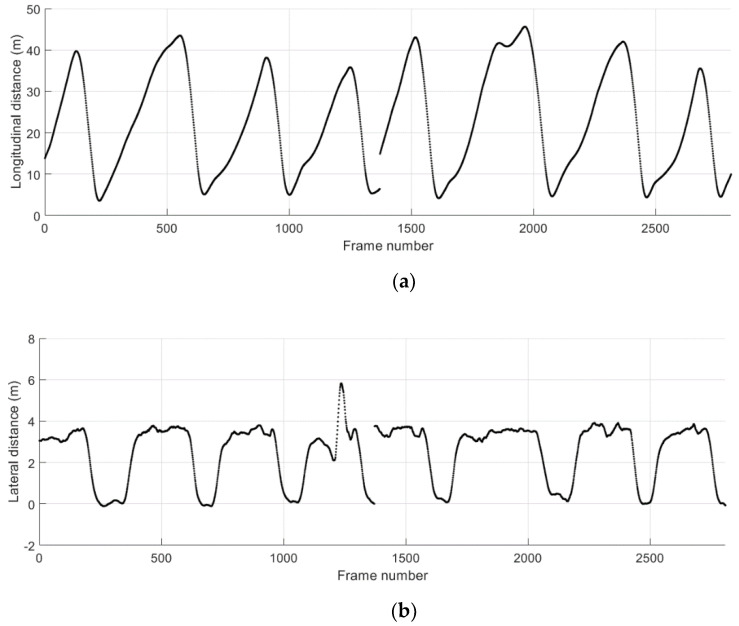

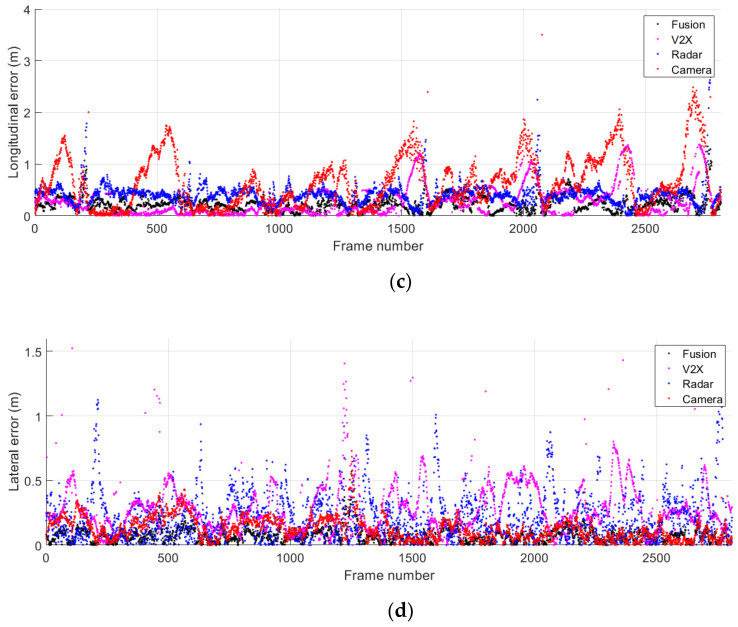
Relative distance to the remote vehicle during the cut-in driving scenario 3. (**a**) Ground truth longitudinal distance; (**b**) ground truth lateral distance; (**c**) absolute error for longitudinal distance estimates; (**d**) absolute error for lateral distance estimates.

**Figure 18:**
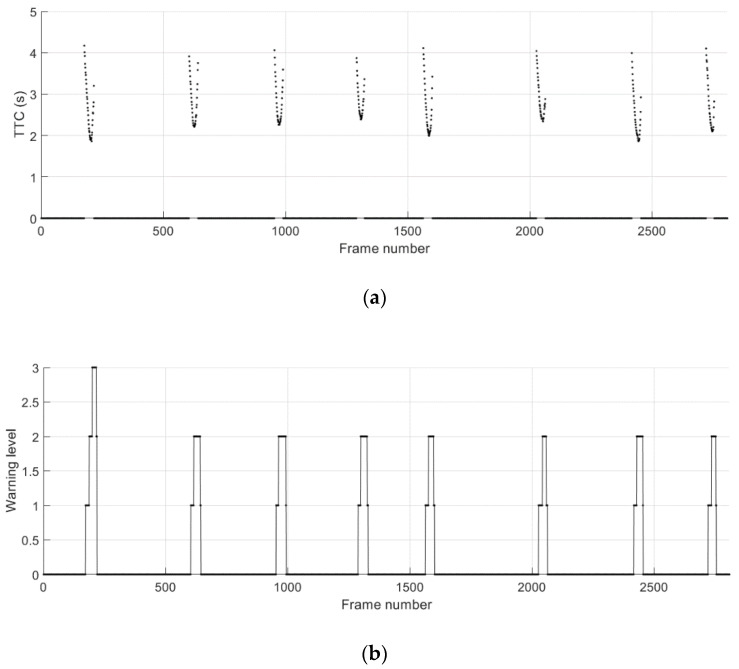
Potential crash events predicted during the cut-in driving scenario 3. (**a**) Time-to-collision (TTC); (**b**) collision warning level.

Figure 17 presents the ground truth relative position of the remote vehicle and the absolute error for the relative position during the cut-in driving scenario 3. The data obtained from separate test runs are concatenated and presented together. A total of eight cut-in events can be recognized in Figure 17b. An unusual remote vehicle maneuver in the lateral direction can be noticed before the initiation of the fourth cut-in maneuver. This was caused by an incident where our driver of the remote vehicle steered away to avoid another vehicle that was used by a different group in the proving ground. Despite the higher fluctuations of the position estimates from the individual remote sensing systems as shown in Figure 17c,d, the proposed cooperative approach provides accurate and reliable positioning results throughout the execution of cut-in maneuvers. The TTC estimates and the corresponding warning levels are presented in Figure 18. The results show that the proposed system for trajectory prediction and risk assessment enables reliable and timely warning to cope with the sudden lane change maneuvers performed in the cut-in driving scenario 3.

## 5. Conclusions

In this paper, we presented the experimental design and performance evaluation of the driving environment perception system based on the fusion of multiple on-board sensors and V2X communications. The two test vehicles used for the driving experiments were each equipped with DSRC equipment and a low-cost GNSS receiver for the exchange of BSM data. The host vehicle was additionally equipped with radar and camera sensors that have already been adopted in production vehicles. The performance of the proposed fusion approach in terms of relative positioning accuracy was evaluated at varying longitudinal and lateral distances between the two test vehicles. In the longitudinal direction, the total RMSE of the relative position estimated with the proposed fusion approach was 0.27 m, whereas those estimated with the camera and radar sensors were 1.91 m and 0.57 m, respectively, which correspond to an improvement of 86% and 52%. In the lateral direction, the total RMSE of the relative position estimated with the proposed method was 0.12 m, while those estimated with the camera and radar were 0.16 m and 0.26 m, respectively, corresponding to an improvement of 27% and 56%. The performance of the trajectory prediction and risk assessment was evaluated in different cut-in driving scenarios where the remote vehicle performs a lane-change maneuver in front of the host vehicle. The test results showed that the proposed method provides accurate target localization and facilitates reliable target trajectory prediction and detection of potential collision during the events when a remote vehicle driving in an adjacent lane cuts in front of the host vehicle.

Although the proposed cooperative approach for driving environment perception proved to be successful in the driving scenarios considered in this study, there still exists a number of challenges to be addressed. The scope of this paper has been limited to driving scenarios where a single remote target is present in the surroundings of the host vehicle. As part of future work, the benefits of the cooperative environment perception approach will be further investigated in scenarios that involve multiple surrounding objects for more diverse use cases. In addition, various factors that could adversely affect the accuracy and reliability of on-board sensors (e.g., high-curvature roads and adverse weather conditions) and V2X communications (e.g., large separations between vehicles, urban environments) will be examined so that the data quality levels that can be expected for each sensor track and for V2X communications can be more accurately determined in continuously changing driving conditions. It is also important to note that the trajectory prediction performed in the scope of this study was based on the physics-based motion model, which performs well for short-term prediction but degrades in its performance when the prediction horizon is extended. Furthermore, this trajectory prediction approach cannot anticipate the state changes caused by varying road curvature, traffic signals, or future driving maneuver execution. Therefore, future work should investigate interaction-aware motion models and also incorporate the map data and the signal phase and timing message, which can be acquired via V2X communications, for accurate and reliable longer-term trajectory prediction that takes into account inter-vehicle interaction, road configuration, and traffic signal dependencies.

## Figures and Tables

**Table 1:** Automotive radar system specifications.

Type	Delphi ESR 2.5	
	Long-Range	Mid-Range
Frequency band	76.5 GHz	76.5 GHz
Range	175 m	60 m
Range accuracy	0.5 m	0.25 m
Angular accuracy	0.5 deg	1.0 deg
Horizontal FOV	20 deg	90 deg
Data update	50 ms	50 ms

**Table 2:** Automotive camera system specifications.

Type	Mobileye 630
Frame size	640 × 480 pixels
Dynamic range	55 dB linear 100 dB in HDR
Range accuracy (longitudinal)	<10% (in general)
Width accuracy	<10%
Horizontal field-of-view (FOV)	38 deg
Data update	66–100 ms

**Table 3:** Dedicated short-range communications (DSRC)-based vehicle-to-everything (V2X) communications characteristics.

Type	IEEE WAVE
Frequency	5.850–5.925 GHz
Channel	1 CCH, 6 SCH
Bandwidth	10 MHz
Data rate	3–27 Mbps
Maximum range	1000 m
Modulation	OFDM
Media access control	CSMA/CA

**Table 4:** Data description for the basic safety message (BSM) core data frame.

Content	Description
Message count	Sequence number for the same type of messages originated from the same sender.
Temporary ID	Device identifier that is modified periodically for on-board units (OBUs). This value may be fixed for roadside units (RSUs).
DSRC second	Milliseconds within a minute that typically represents the moment when the position was determined.
Position	Geographic latitude, longitude, and height.
Position accuracy	Semi-major axis (length and orientation) and semi-minor axis (length) of an ellipsoid representing the position accuracy.
Transmission state	Vehicle transmission state (i.e., neutral, park, forward, and reverse).
Speed	Vehicle speed.
Heading	Vehicle heading. Past values may be used if the sender is stopped.
Steering wheel angle	Angle of the vehicle steering wheel.
Acceleration	Vehicle acceleration in longitudinal, lateral, and vertical axes.
Yaw rate	Vehicle yaw rate.
Brake system status	Status of the brake and other control systems (i.e., traction control, ABS, stability control, brake boost, and auxiliary brake).
Vehicle size	Vehicle width and length.

**Table 5:** Vehicle collision warning conditions [23].

Condition	Stage	Warning Type	**Color**
No collision detected	No threat (Level 0)	Visual	Gray
TTC>2.6 s	Threat detected (Level 1)	Visual	Green
1.6 s<TTC≤2.6 s	Inform driver (Level 2)	Visual and audible	Yellow
TTC≤1.6 s	Warn driver (Level 3)	Visual and audible	Red

**Table 6:** Normal driving scenario descriptions.

Scenario Number	Host Vehicle Speed (km/h)	Remote Vehicle Speed (km/h)	Remote Vehicle Driving Lane
1	20	25	Same as HV
2	20	25	Adjacent to HV

**Table 7:** Relative positioning accuracy in the longitudinal direction for the measurements obtained during the normal driving scenario 1.

Data Range (m)	Camera Lon. Position Error		Radar Lon. Position Error		V2X Lon. Position Error		Fusion Lon. Position Error	
	RMSE (m)	SD (m)	RMSE (m)	SD (m)	RMSE (m)	SD (m)	RMSE (m)	SD (m)
0–10	0.36	0.24	0.39	0.18	0.25	0.22	0.13	0.13
10–20	0.19	0.15	0.51	0.21	0.31	0.28	0.12	0.11
20–30	0.33	0.23	0.54	0.15	0.38	0.37	0.24	0.09
30–40	0.63	0.45	0.56	0.20	0.33	0.33	0.29	0.16
40–50	1.23	0.79	0.74	0.42	0.40	0.38	0.24	0.23
50–60	1.82	0.98	1.09	0.57	0.33	0.33	0.23	0.23
60–70	2.73	1.69	0.83	0.32	0.26	0.25	0.28	0.16
Total	1.16	0.96	0.67	0.35	0.34	0.32	0.22	0.18

**Table 8:** Relative positioning accuracy in the lateral direction for the measurements obtained during the normal driving scenario 1.

Data Range (m)	Camera Lat. Position Error		Radar Lat. Position Error		V2X Lat. Position Error		Fusion Lat. Position Error	
	RMSE (m)	SD (m)	RMSE (m)	SD (m)	RMSE (m)	SD (m)	RMSE (m)	SD (m)
0–10	0.05	0.05	0.21	0.21	0.12	0.09	0.05	0.04
10–20	0.05	0.04	0.21	0.16	0.24	0.10	0.06	0.04
20–30	0.06	0.06	0.26	0.20	0.32	0.13	0.09	0.06
30–40	0.07	0.07	0.24	0.19	0.45	0.16	0.14	0.08
40–50	0.11	0.10	0.28	0.28	0.62	0.20	0.19	0.11
50–60	0.12	0.12	0.34	0.29	0.70	0.31	0.20	0.10
60–70	0.15	0.15	0.28	0.28	0.84	0.41	0.19	0.12
Total	0.09	0.09	0.26	0.23	0.48	0.28	0.13	0.09

**Table 9:** Relative positioning accuracy in the longitudinal direction for the measurements obtained during the normal driving scenario 2.

Data Range (m)	Camera Lon. Position Error		Radar Lon. Position Error		V2X Lon. Position Error		Fusion Lon. Position Error	
	RMSE (m)	SD (m)	RMSE (m)	SD (m)	RMSE (m)	SD (m)	RMSE (m)	SD (m)
0–10	N/A	N/A	0.30	0.28	0.44	0.40	0.31	0.31
10–20	0.49	0.48	0.40	0.31	0.36	0.36	0.34	0.32
20–30	0.91	0.80	0.44	0.39	0.41	0.40	0.40	0.40
30–40	1.52	1.37	0.43	0.40	0.47	0.46	0.42	0.42
40–50	2.80	1.82	0.37	0.33	0.34	0.33	0.31	0.30
50–60	3.61	2.52	0.39	0.26	0.27	0.26	0.24	0.22
60–70	4.29	3.50	0.31	0.26	0.38	0.33	0.28	0.27
Total	2.62	2.20	0.39	0.32	0.37	0.37	0.33	0.32

**Table 10:** Relative positioning accuracy in the lateral direction for the measurements obtained during the normal driving scenario 2.

Data Range (m)	Camera Lat. Position Error		Radar Lat. Position Error		V2X Lat. Position Error		Fusion Lat. Position Error	
	RMSE (m)	SD (m)	RMSE (m)	SD (m)	RMSE (m)	SD (m)	RMSE (m)	SD (m)
0–10	N/A	N/A	0.32	0.22	0.15	0.09	0.11	0.09
10–20	0.15	0.07	0.29	0.18	0.18	0.06	0.05	0.05
20–30	0.16	0.08	0.28	0.25	0.31	0.15	0.09	0.08
30–40	0.17	0.11	0.25	0.24	0.42	0.19	0.07	0.06
40–50	0.28	0.14	0.25	0.25	0.57	0.20	0.07	0.07
50–60	0.27	0.17	0.26	0.26	0.68	0.30	0.12	0.12
60–70	0.26	0.17	0.25	0.24	0.79	0.32	0.09	0.09
Total	0.22	0.14	0.27	0.26	0.51	0.30	0.09	0.09

**Table 11:** Cut-in driving scenario descriptions.

Scenario Number	Host Vehicle Speed (km/h)	Remote Vehicle Speed (km/h)	Cut-In Distance (m)	Number of Cut-In Maneuvers
1	40–45	35–40	15–20	4
2	40–45	25–30	15–20	5
3	40–45	15–20	15–20	8

## Data Availability

Not applicable.

## Abbreviations

ADAS	Advanced driver assistance system
BSM	Basic safety message
CAM	Cooperative awareness message
CAN	Controller area network
CAV	Connected and automated vehicle
CTRV	Constant turn rate and velocity
C-V2X	Cellular vehicle-to-everything
DENM	Decentralized environmental notification message
DSRC	Dedicated short-range communications
ECU	Electronic control unit
FMCW	Frequency modulated continuous wave
FOV	Field of view
GNSS	Global navigation satellite system
ITS	Intelligent transportation system
NTRIP	Networked Transport of RTCM via Internet Protocol
OBU	On-board unit
ODD	Operational design domain
RSU	Roadside unit
RTCM	Radio Technical Commission for Maritime Services
TTC	Time-to-collision
V2I	Vehicle-to-infrastructure
V2N	Vehicle-to-network
V2P	Vehicle-to-pedestrian
V2V	Vehicle-to-vehicle
V2X	Vehicle-to-everything
WAVE	Wireless access in vehicular environments
WGS	World Geodetic System
